# YODA: Software to facilitate high-throughput analysis of chronological life span, growth rate, and survival in budding yeast

**DOI:** 10.1186/1471-2105-11-141

**Published:** 2010-03-18

**Authors:** Brady Olsen, Christopher J Murakami, Matt Kaeberlein

**Affiliations:** 1 Department of Pathology, University of Washington, Seattle, WA 98195, USA; 2 Institute of Aging Research, Guangdong Medical College, Dongguan 523808, China

## Abstract

**Background:**

The budding yeast *Saccharomyces cerevisiae *is one of the most widely studied model organisms in aging-related science. Although several genetic modifiers of yeast longevity have been identified, the utility of this system for longevity studies has been limited by a lack of high-throughput assays for quantitatively measuring survival of individual yeast cells during aging.

**Results:**

Here we describe the Yeast Outgrowth Data Analyzer (YODA), an automated system for analyzing population survival of yeast cells based on the kinetics of outgrowth measured by optical density over time. YODA has been designed specifically for quantification of yeast chronological life span, but can also be used to quantify growth rate and survival of yeast cells in response to a variety of different conditions, including temperature, nutritional composition of the growth media, and chemical treatments. YODA is optimized for use with a Bioscreen C MBR shaker/incubator/plate reader, but is also amenable to use with any standard plate reader or spectrophotometer.

**Conclusions:**

We estimate that use of YODA as described here reduces the effort and resources required to measure chronological life span and analyze the resulting data by at least 15-fold.

## Background

The ability to accurately monitor survival and growth rate of cells is essential for many assays employed in studies of the budding yeast. Changes in growth rate and survival over time are often monitored in response to a chemical treatment, environmental change (e.g. temperature, starvation, etc.), or genetic variant. For example, the yeast ORF deletion collection, which consists of >5000 unique single-gene deletion strains in an isogenic background, has been queried for more than 100 unique phenotypes by monitoring growth or viability under different conditions [1]. Growth rate (doubling time) of yeast cells can be quantified by monitoring the change in optical density at 600 nm (OD_600_) of a yeast culture under specified conditions. Survival of yeast cells has traditionally been quantified by plating the cells onto rich growth medium (yeast peptone dextrose, YPD) and counting colony forming units (CFUs) before and after treatment.

One important assay that involves monitoring survival of yeast cells over time is measurement of chronological life span (CLS), which is defined as the length of time a yeast cell is able to maintain viability during post-diauxic growth arrest [2]. Yeast CLS has emerged as a useful paradigm in aging-related research and has led to the identification of several dozen genetic modifiers of yeast longevity, some of which play a conserved aging-related function in multicellular eukaryotes [3]. CLS has traditionally been performed by culturing cells in a synthetically defined medium and monitoring survival over time (every 2-3 days) by periodically removing an aliquot of the aging culture, serially diluting that aliquot, plating the cells onto YPD and counting CFUs [4].

We have recently developed a high-throughput method of measuring CLS that involves quantifying survival based on measuring the outgrowth of a defined number of cells by monitoring OD_420-580 _using a combined shaker/incubator/plate reader, the Bioscreen C MBR machine (Growth Curves USA) [5,6]. This method is based on the fact that the optical density of a culture after a fixed period of growth will be proportionate to the number of viable cells present in the culture initially. We have used a qualitative version of this method in which only a single outgrowth optical density measurement was taken to screen the yeast ORF deletion collection for long-lived single-gene deletion strains [7], and more recently, the quantitative Bioscreen C MBR method has been used to quantify the effects of media composition on CLS, and to define a molecular mechanism of chronological aging in yeast [8,9]. Briefly, this method involves culturing cells in individual 5 mL cultures in tubes on a rotating drum at 30°C (the aging cultures). At each age-point (e.g. day 2, 4, 6, etc.), 5 μL of each aging culture is inoculated into 145 μL of YPD in one well of a Bioscreen Honeycomb plate. Outgrowth of the cells from each aging culture is then determined by taking OD_420-580 _measurements for each well every 30 minutes for 24 hours using the Bioscreen C MBR machine. The Bioscreen C MBR machine has a maximum capacity of two 100-well Honeycomb plates, allowing for simultaneous measurement of outgrowth for up to 200 individual aging cultures. Survival at each age-point is determined by quantifying the rightward shift of the outgrowth curve along the time axis for each aging culture relative to the outgrowth curve for the aging culture at the initial age-point (day 2 of culture) using the formula:

where s_n _is the survival percentage, Δt_n _is the time shift, and δ_n _is the doubling time. Detailed methodology and a video protocol describing the CLS assay have been published, and we refer the interested reader to these references [5,6].

In order to automate analysis of the data generated during a CLS experiment, we developed a software package called YODA, the Yeast Outgrowth Data Analyzer. YODA accepts as input single or multiple text files containing OD values as a function of time (one file for each age-point) and returns several useful parameters, including maximal growth rate for each well, survival at each age-point for each aging culture, and the survival integral (SI) for each strain, which is defined as the area under the survival curve. YODA also has the capacity to group data from replicate cultures and perform simple statistical analyses of each group of replicates both individually and relative to experiment-matched control replicates. YODA is provided to the scientific community as a freely available utility on our website at http://www.kaeberleinlab.org/yoda and on the SAGEWEB website at http://www.sageweb.org/yoda. We have also provided the YODA source code for download along with an issue tracker at http://code.google.com/p/sageweb-yoda/.

Here we describe the key features of YODA and provide a demonstration of how YODA can be used to analyze data from a typical CLS experiment. We also provide examples of how YODA can be used for additional types of experiments in yeast where quantitation of cell survival or growth rate are desired and describe how data generated from sources other than a Bioscreen C MBR machine can be analyzed with YODA.

## Implementation

### Uploading outgrowth data from a CLS experiment

Detailed instructions for performing chronological aging experiments using the Bioscreen C MBR machine are published [5,6] and are also available on our website. At each age-point, the Bioscreen "EZExperiment" software produces a comma-delimited file containing OD_420-580 _values for each of the 200 wells corresponding to the maximal loading capacity of the machine (2 × 100 well plates). The first column of the resulting table contains the "Time" data (30 minute intervals by our protocol [5,6], although this can be varied) and each subsequent column contains the data for a single well. In order to upload your data into YODA, the outgrowth data for each age-point should be saved in a separate comma-delimited (.csv) or Microsoft Excel (.xls) file. It is important that well position be maintained for each aging culture throughout the entire experiment (e.g. wild type replicate #1 always in well #1, mutant A replicate #1 always in well #2, wild type replicate #2 always in well #3, etc.). This is ensured by loading the wells in the same order at each age-point. Save each file with an appropriate identifying name, such as 'name_date.csv'.

**Figure 1:**
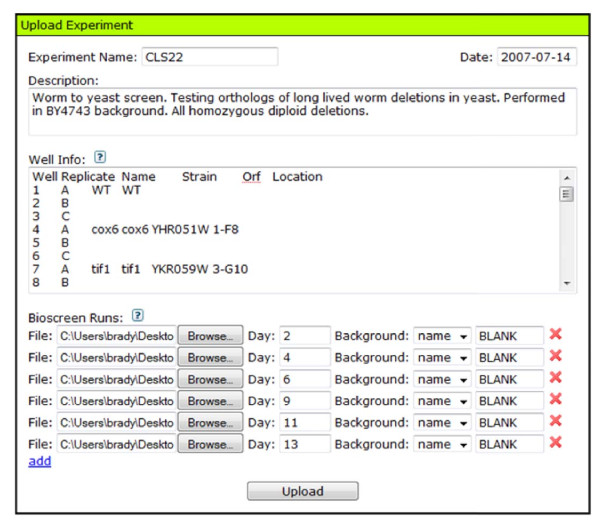
**Upload experiment form**. Set up an experiment's well info (position, name, group, etc.) and input files. Select an age-point for each file and a background well or value to be subtracted from each OD reading in the corresponding input file.

Once your files are formatted and named appropriately, they can be uploaded to YODA by choosing the 'Upload' link in the Analyzer Menu. This will display the Upload Experiment form (Fig. 1). Fill in the experiment name, start date, and description of your experiment. If you want to use named wells for grouping, fill in the well info input (click the question mark next to the Well Info text box on the Upload Experiment form for detailed information about the format).

**Figure 2:**
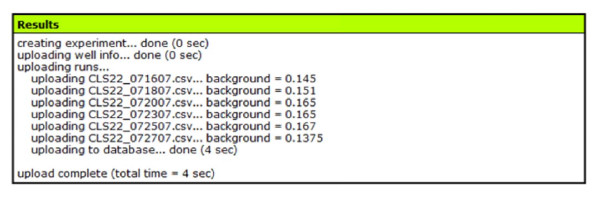
**Upload results window showing runs and backgrounds**.

Upload each of your age-point outgrowth data files. Assign each run a day (for example, corresponding to the age of the culture at that age-point) so that YODA can determine which point in the survival curve belongs to which run (survival curve integrals automatically take into consideration days between runs). If you have named your wells using the well info input, type the name of the media-only well in the background field to compute a background reading for the run. The background reading defines the optical density value for a well with media only. Otherwise, select 'value' from the background type drop-down and type in a custom background (the default is 0.15, which is approximately the OD reading for 150 μL of YPD in the Bioscreen). When all of the data files for the experiment have been selected, click the 'Upload' button. YODA will tell you what the computed backgrounds are and if any errors have occurred in the results window (Fig. 2).

### Quantifying growth rate and survival with YODA

**Figure 3:**
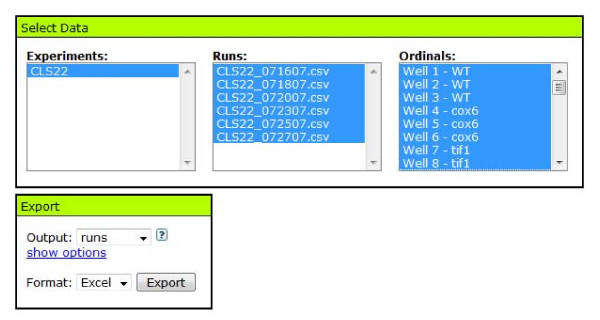
**Export form**. Select files and wells to export.

To analyze your CLS data and export it for graphical presentation, click the "Export" link in the Analyzer Menu. This will display the Export form (Fig. 3). Select the desired experiment, data sets (age-points), and well positions. YODA's default settings will select all data sets and well positions (1-200) for a particular experiment, unless otherwise selected.

**Figure 4:**
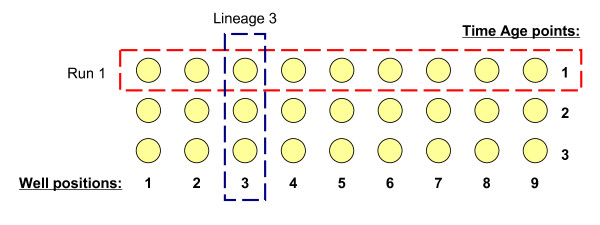
**Exporting runs and lineages**. "Runs" gives information about each well independent of separate age-points (such as OD readings and doubling time). "Lineages" gives information about a well position across multiple age-points (such as time shift and survival integral).

Under the Export window, set the "output" drop-down to either "runs" or "lineages" (Fig. 4). By default, selecting 'runs' will output well name, run name, run day, doubling times, and background normalized OD_420-580 _readings (click the "show options" link to adjust what parameters are exported and how they are calculated. See Table 1). To export the OD_420-580 _readings for a single well position across all data sets for a given experiment, select just the desired well in the select box and set the output drop-down to 'runs'. Selecting "lineages" will output doubling time, survival values at each age-point, and survival integrals for each well across age-points. If replicates have been grouped using the "Well Info" option, the average or median values for a well group can be output by selecting the desired option under "grouping". In order to calculate survival values at each age-point, the first age-point is defined as maximal survival (1.0) and subsequent survival values are calculated as the relative fraction surviving at each age-point. To graphically present survival curves from YODA, plot the fractional survival (y-axis) as a function of age (x-axis) for each well or replicate.

**Table 1:** YODA export output options.

Grouping Options:	
none	Outputs info for each well or well position.
average groups	Outputs average value and standard deviation for each well group described in well info.
median groups	Outputs median value and standard deviation for each well group described in well info.

**Curve Parameter Options:**	

OD thresholds	The min and max background normalized OD values to use in doubling time calculation.
Doubling time Interval OD	The min and max background normalized OD values to calculate "doubling time interval". The program will calculate the average doubling time over this interval.
Survival time shift OD	The background normalized OD value to calculate time shift between curves at different age points.

**Run Export Options:**	

append ODs	Whether or not the OD readings for a well should be appended to output.
subtract background	Outputs the OD readings with the file's background subtracted
doubling time inflection	Outputs the doubling time calculated where the curve has the steepest slope between min and max OD thresholds.
doubling time interval	Outputs the average doubling time calculated at all points between min and max doubling time threshold ODs.
doubling time correction	Outputs empirically corrected doubling times.

**Lineage Export Options:**	

survival	Outputs the survival fractions for each age point.
survival area	Outputs the integral of the survival fractions over all age points.
clean	Survival fractions are cleaned so that the survival curve has no increases or gasping (spikes at end).
show time shifts	Outputs the time shift relative to reference curve (first age point).
doubling time method	The doubling time method used to calculate survival fraction (uses doubling time of first age point curve).
% change versus reference	Calculates the percent change in survival integral of each well versus a reference.
log2 ratio versus reference	Calculates the log base 2 ratio of survival integrals of each well versus a reference.
t-test versus reference	Calculates the t-statistic and p-value for each well group versus a reference (grouping option must be selected).

### Using YODA for non-aging assays

YODA can be easily adapted for analysis of data for a variety of assays in which growth rate or survival is the parameter of interest. To quantify growth rate from OD_420-580 _readings obtained with a Bioscreen C MBR machine, simply upload a single comma-delimited "EZExperiment" output file as described above and export the data using the "runs" option from the "output" drop-down menu under the Export window. Quantification of survival in response to different experimental stimuli can be performed in a manner analogous to that described for CLS above, with the control treatment assigned as the first age-point in the CLS experiment and each experimental group assigned as a subsequent age-point. Specific examples are provided in the **Results **section below.

### Using YODA with alternative methods of optical density determination

Although YODA is designed for simplified analysis of OD data obtained from the Bioscreen C MBR machine, OD measurements obtained from alternative sources, such as a standard plate reader or spectrophotometer, can also be analyzed with YODA. For such uses, simply ensure that the OD data is entered into .csv or .xls files in the manner described above for data obtained from the Bioscreen machine. All subsequent steps in the analysis are identical.

## Results

### Quantitation of yeast CLS with YODA

**Figure 5:**
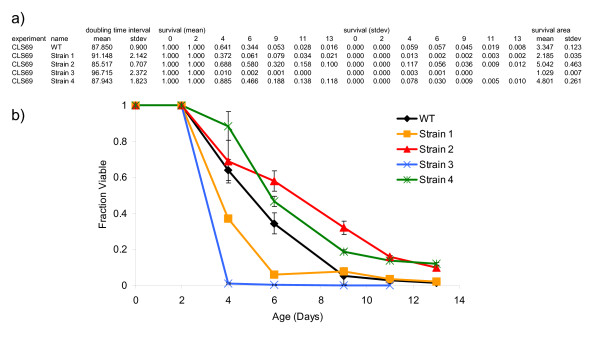
**YODA CLS output and survival curves**. CLS results from one control strain (BY4743) and four single gene deletions in the same background. A) Output from YODA using the parameters described. Strain name, doubling times, survival at each time-point, and survival integrals with standard deviations are shown. B) Survival curves associated with the data output from YODA. Error bars represent the standard deviation of three biological replicates.

In this exemplary yeast CLS experiment, the aging potential of a control and four single-gene deletion mutant strains was determined using a Bioscreen C MBR machine. Outgrowth analysis was performed as described above by inoculating 5 μL of each aging culture into 145 μL of YPD in individual wells of a Bioscreen Honeycomb plate. Each strain was assayed in triplicate using biological replicates (3 independently derived aging cultures per genotype). Outgrowth was determined at the following age-points: days 2, 4, 6, 9, 11, and 13. Outgrowth data for each age-point was retrieved as comma-delimited text files from the EZExperiment software (Additional File 1) and uploaded to YODA as described above. 'Average groups' was selected under the 'Grouping' options in the Export screen of YODA and the data was exported as a Microsoft Excel Worksheet. Survival curves were plotted from the data generated by YODA along with the standard deviation at each age-point for each strain (Fig. 5).

### Quantitation of yeast survival following heat shock

In addition to determining the CLS of yeast strains, YODA can also be used to quantify cell viability following an environmental stressor such as heat shock. As an example experiment, an overnight culture of wild type cells was split into 100 μL aliquots and subjected to varying lengths of 55°C heat shock (0, 5, 10, 15, and 20 minutes). Following heat shock, 5 μL of each treatment was inoculated into 145 μL YPD in individual wells of a Bioscreen Honeycomb plate. Each treatment was assayed in triplicate.

**Figure 6:**
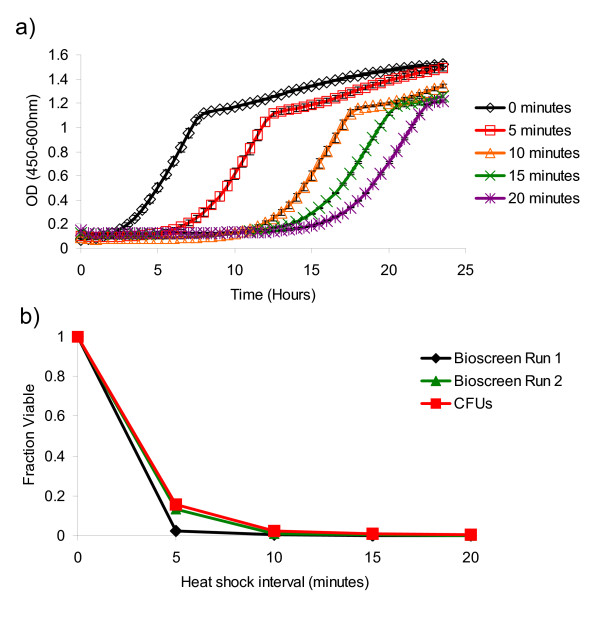
**Determining loss of viability due to heat shock using YODA**. BY4742 cells show a loss in viability following 55°C heat shock. A) Average growth curves from three technical replicates are shown for each heat shock treatment. B) Survival curves from two independent Bioscreen runs as well as CFUs. All experiments assume the 0 minute treatment to have 100% cell viability.

To quantify viability following heat shock, the data output by the Bioscreen was split into individual comma-delimited (.csv) files, based on the length of time the cells were subjected to heat shock. For example, the first three wells of the Bioscreen plate were inoculated with cells subjected to 0 minutes of heat shock, the fourth through sixth wells were inoculated with cells subjected to 5 minutes of heat shock, and so forth. The data from the first three wells were put into a new comma-delimited file along with the data from one blank well; the data from the fourth through sixth wells were put into a second comma delimited file along with the data from the same blank well, and so on until all treatments were accounted for. Once each treatment had been placed into a separate .csv file, all files were then uploaded to YODA (see Additional File 2, for the original Bioscreen output file and the modified .csv files loaded into YODA). In the 'Upload Experiment' form, "0 minutes, 5 minutes, 10 minutes, etc." were filled in the 'Day' field next to the corresponding .csv files. 'Average groups' was selected under the 'Grouping' options in the Export screen of YODA and the data was exported as a Microsoft Excel Worksheet. Outgrowth and survival curves were generated from two independent experiments (Fig. 6). For comparison, viability following heat shock was also quantified using the traditional labor-intensive method of plating cells onto solid media following serial dilution and counting CFUs. CFU counts showed a similar loss in viability due to heat shock as was observed using the Bioscreen.

### Quantitation of growth inhibition by rapamycin

**Figure 7:**
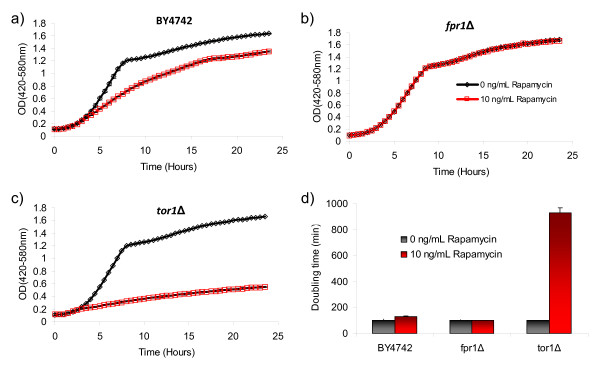
**Determining rapamycin sensitivity using YODA**. Cells from A) BY4742, B) *fpr1Δ*, and C) *tor1Δ *overnight cultures were inoculated into YPD or YPD containing 10 ng/mL rapamycin. Average growth curves from three technical replicates are shown along with D) doubling times generated by YODA. The "interval" method was used to calculate doubling times.

As an alternative to survival, it is often desirable to monitor changes in growth kinetics in response to a stress or stimulus. To demonstrate how to use YODA for these types of assays, we examined the response of wild type, *tor1Δ*, and *fpr1Δ *cells to rapamycin, a chemical inhibitor of the target of rapamycin kinase and a putative anti-aging compound [10,11]. For this experiment, cells were grown overnight in YPD and 5 μL of each culture was inoculated into either 145 μL of YPD or YPD containing 10 ng/mL rapamycin in individual Honeycomb plate wells. Each strain was assayed using five technical replicates. Outgrowth data was obtained using the Bioscreen C MBR machine, as described above. The resulting comma-delimited text file was uploaded to YODA with 'average groups' selected under 'grouping' as well as 'media' under the 'append properties' category. Growth curves were plotted using the data generated by YODA in Microsoft Excel (Fig. 7). As predicted from previously published data [12], the *tor1Δ *strain was sensitive to rapamycin, while the *fpr1Δ *strain was resistant.

## Conclusions

YODA provides a web-based platform for quantifying survival and growth inhibition in the budding yeast. YODA was designed to facilitate high-throughput studies of yeast CLS, but is equally suitable for identifying genetic variants or environmental interventions that modify cell survival or growth rate. In addition to providing information not attainable using the traditional CFU method, such as growth rate of each strain, the use of YODA with a machine such as the Bioscreen C MBR enhances efficiency in the laboratory. For example, the method described here can accommodate up to three biological replicates of 66 strains in a single experiment (198 out of 200 wells used). We estimate that an entire experiment of this nature requires approximately 4 hours of effort for an experienced researcher, including preparation time, strain handling, and data analysis. In contrast, we estimate that the traditional CFU method with serial dilutions would require at least 15-fold longer for the same researcher to obtain equivalent data. In terms of resources, both methods require equal volumes of media for aging cultures; however, the method described here requires only 30 mL of liquid media and two multi-well plates per age point, whereas the traditional CFU method requires approximately 5 L of agar-based media and 198 Petri dishes per age-point (25 mL per plate × 198 strains). Thus we conclude that YODA in combination with the methods described here results in a significant reduction in time and resources required to measure CLS or perform other survival-based assays in yeast.

## Availability and Requirements

YODA is provided to the scientific community as a freely available utility on our website at http://www.kaeberleinlab.org/yoda and on the SAGEWEB website at http://www.sageweb.org/yoda. YODA can be used anonymously without a username and password. We have also provided the YODA source code for download along with an issue tracker at http://code.google.com/p/sageweb-yoda/. Installation requires a web server with PHP 5.2 support and MySQL 5.1 or above.

## Authors' contributions

BO, CJM, and MK conceived of the project. MK wrote the manuscript. BO developed the software. CJM performed the yeast experiments. All authors read and approved the final manuscript.

## Supplementary Material

Additional file 1 **Outgrowth data for yeast chronological lifespan quantitation**. This zip contains files formatted for YODA for chronological lifespan quantitation. Files Day2.csv, Day4.csv, Day6.csv, Day9.csv, Day11.csv, and Day13.csv serve as inputs for "runs" on the YODA upload page. Each is a comma-delimited OD measurement file for aging cultures at a given age-point. The first column contains OD measurement times in HH:MM:SS format (H = hours, M = minutes, S = seconds). All other columns holds outgrowth curve ODs at the corresponding time-point for each well in the plate. The naming_convention.csv file contains the input for "well info" under the YODA upload page. Click here for file

Additional file 2 **Outgrowth data for heat shock survival quantitation**. This zip contains files formatted for YODA for heat shock survival quantitation. Files heat_shock_0min.csv to heat_shock_30min.csv serve as inputs for "runs" on the YODA upload page to calculate time shift and relative survival with increasing heat shock duration. Each is a comma-delimited OD measurement file under various magnitudes of heat shock. The first column contains OD measurement times in HH:MM:SS format (H = hours, M = minutes, S = seconds). All other columns holds outgrowth curve ODs at the corresponding time-point for each well in the plate. The heat_shock_output.csv file contains combined OD readings from heat_shock_0min.csv to heat_shock_30min.csv. The naming_convention.csv file contains the input for "well info" under the YODA upload page for heat_shock_output.csv. Click here for file
